# Model of the Magnetostrictive Hysteresis Loop with Local Maximum

**DOI:** 10.3390/ma12010105

**Published:** 2018-12-30

**Authors:** Roman Szewczyk

**Affiliations:** Institute of Metrology and Biomedical Engineering, Warsaw University of Technology, 02-525 Warsaw, Poland; szewczyk@mchtr.pw.edu.pl; Tel.: +48-609-464741

**Keywords:** magnetostriction model, Mn-Zn ferrites, construction steels

## Abstract

This paper presents a model of the magnetostrictive hysteresis loop with local maximum. The model is based on the differential equations describing magnetostriction due to the domain wall movement as well as domain magnetization rotation. The transition between these mechanisms of magnetization is quantified by the Maxwell–Boltzmann distribution. Moreover, the lift-off phenomenon in the magnetostrictive hysteresis loop is considered. The proposed model was validated on the results of measurements of magnetostrictive hysteresis loops of Mn_0.70_Zn_0.24_Fe_2.06_O_4_ ferrite for power application and 13CrMo4-5 construction steel. The results of modeling confirm that the proposed model corresponds well with experimental results. Good agreement was confirmed by determination coefficient R^2^, which exceeded 0.995 and 0.985 for Mn_0.70_Zn_0.24_Fe_2.06_O_4_ ferrite for power application and 13CrMo4-5 construction steel, respectively.

## 1. Introduction

The magnetostriction phenomenon is connected with the changes of the linear dimensions of the sample during the magnetization process. The magnetostrictive phenomenon has great technical importance. It can be used in the development of specialized position [1] and level sensors [2], MEMS (Micro-Electro-Mechanical Systems) sensors [3], as well as ultrasonic transducers [4] and high accuracy actuators [5].

In spite of the fact that magnetostriction has been known for over one hundred years, as well as the great effort that has been taken to understand it, until now a complete quantitative model of the magnetostrictive hysteresis loop has not been presented. However, it is obvious that magnetostrictive characteristics of soft magnetic materials plays a key role in understanding other magnetomechanical effects, such as the Villari effect [6], the Wiedemann effect [7], or the stress dependence of giant magnetoimpedance phenomenon [8], as well as the Matteucci effect [9].

Previously presented research clearly indicates that magnetostriction (*λ*) is mostly determined by a parabola-shaped curve with the value of magnetization (*M*) of the material (which may be estimated by the flux density (*B*)) [10]. Another important approach was the use of hyperbolic Bessel functions, proposed by Sablik [11]. However, proposed solutions are suitable for parabola-shaped *λ*(*B*) curves only. It should be stressed that alarge number of soft magnetic materials, such as some Mn-Zn ferrites [12] or constructional steels [13,14], exhibit local maxima on the *λ*(*B*) dependence, which makes parabola-shaped models unsuitable for the modeling of magnetostrictive hysteresis loops of such materials.

This paper fills the gap in the state of the art, presenting a quantitative model of the magnetostrictive *λ*(*B*) hysteresis loop with local maximum. The presented model considers the transition from the domain wall movement to the domain rotations, causing the changes of the sign of magnetostriction curve derivative. As a result, the proposed model may be used for both the technical development of magnetostrictive sensors and actuators, as well as for physical analyses of magneto-mechanical processes in soft magnetic materials.

## 2. Principles of Modeling the Magnetostrictive Hysteresis Loops

Magnetostriction in soft magnetic materials is caused by the changes of the total free magneto-mechanical energy of the material sample due to the transition from the paramagnetic to ferromagnetic state, during the cooling of the material and overturning of the Curie temperature. On the basis of magneto-crystalline anisotropy analyses, it may be stated that the dependence of magnetostriction (*λ*) on the magnetization (*M*) of magnetic material may be described by the fourth order polynomial [15]:

(1) $$\lambda \left(M\right)=a_{1}M^{2}+a_{2}M^{4}$$

This dependence is commonly reduced to the second order polynomial [16]:

(2) $$\lambda \left(M\right)=\frac{3}{2}\frac{\lambda_{s}}{{M_{s}}^{2}}M^{2}$$

where *λ_s_* is the saturation magnetostriction and *M_s_* is saturation magnetization. In the case of soft magnetic materials, where relative permeability *μr* >> 1, Equation (2) may be presented as [17]:

(3) $$\lambda \left(B\right)=\frac{3}{2}\frac{\lambda_{s}}{{B_{s}}^{2}}B^{2}$$

where *B* and *B_s_* are flux density in the material and saturation flux density of the material, respectively. Such form of magnetostrictive characteristic model is more convenient for technical applications. For this reason, Equation (3) was successfully used for the technical modeling of magnetostrictive actuators, especially with cores made of giant magnetostrictive materials, such as Terfenol-D [18,19].

On the other hand, analysis of experimental results of measurements of *λ*(*B*) hysteresis loop clearly indicates that accurate modeling of these characteristics requires consideration of *λ*(*B*) hysteresis (which is different than *B*(*H*) hysteresis), as well as the so called “lift-off” phenomenon. The “lift-off” phenomenon is connected with the fact that, during the magnetization loop, magnetostriction never comes back to the value observed in the demagnetized state.

The physical origins of hysteresis on the *λ*(*B*) relation are connected with the interaction between residual stresses and magnetostrictive strain. In previous research, it was connected with the hysteretic magnetization equal to the difference between total magnetization (*M*) and anhysteretic magnetization (*M_an_*) [20]. Another approach to this hysteresis was based on the hyperbolic Bessel functions [11], connecting the magnetostriction with the efficient magnetizing field (*H_e_*) in the magnetic material equal:

(4) $$H_{e}=H+\alpha M$$

where *H* is the magnetizing field, whereas *α* is the interdomain coupling accordingly to the Bloch theory.

The “lift-off” phenomenon is connected with the fact that, during the magnetization process, the *λ*(*B*) characteristic never returns to zero [20]. The physical origins of this effect are not clearly explained; however, it has been observed in experimental measurements of the magnetostrictive hysteresis loops *λ*(*B*) and *λ*(*H*) of most ferromagnetic materials [11,20,21,22,23,24]. Known previous approaches to quantitative modeling of the “lift-off” phenomenon were focused on the reproduction of the shape of magnetostrictive hysteresis loops considering this phenomenon [20].

The most important problem connected with modeling the magnetostrictive hysteresis loops *λ*(*B*) and *λ*(*H*) is the fact, in the case of some soft magnetic materials, that local maxima occurs on these dependences. This local maxima was observed in experimental results [12,14,25,26,27]; however, the quantitative model of such a magnetostrictive hysteresis loop was never presented before. Lack of such a model is the significant barrier for understanding the physical background of magneto-mechanical effects, as well as for the practical description of the behavior of ferromagnetic materials required for, for example, the development of transformers or nondestructive testing of elements made of constructional steels.

## 3. The Proposed Model of the Magnetostrictive Hysteresis Loop with Local Maxima

The proposed model of the magnetostrictive *λ*(*B*) hysteresis loop is based on the fact that the mechanism of the magnetization of ferromagnetic material changes during the magnetization process. For smaller values of magnetizing field, magnetization is connected with the domain walls movements, whereas magnetization in the saturation region is mostly caused by the rotation of domains magnetization [15,16]. As a result, magnetostriction *λ_mov_*(*B*) in the domain walls movement region of the magnetic hysteresis loop has a parabola shape [16], and may be described by the following differential equation:

(5) $$\frac{d\lambda_{mov}}{dB}=2a_{1}B$$

where *a*_1_ is the parameter determined in the same way as the parameters in Equation (3). However, for the domain rotations region, the magnetostriction *λ_rot_* is connected mostly with the rotation from the easy axis to hard axis. As a result, the linear dependence of *λ_rot_*(*B*) may be observed in this area [28], represented by the following differential equation:

(6) $$\frac{d\lambda_{rot}}{dB}=a_{2}$$

where *a*_2_ is the parameter describing the slope of the magnetostrictive curve in the saturation region. The transition between the magnetization mechanisms based on the domain wall movement and domain magnetization rotation may be quantified by the Maxwell–Boltzmann statistical distribution, of which the cumulative distribution function is given by the following equation [29]:

(7) $$W\left(B\right)=erf\left(\frac{B-B_{switch}}{k\sqrt{2}}\right)-\sqrt{\frac{2}{\pi }}\frac{\left(B-B_{switch}\right)e^{-\left(B-B_{switch}\right)/\left(2k^{2}\right)}}{k}$$

where *B_switch_* is the value of flux density *B* when the mechanism of magnetization starts to change from domain walls movement to domain magnetization rotation, and *k* determines the intensity of this process. The so-called error function *erf*(*x*), necessary to determine Maxwell­­–Boltzmann statistical distribution, is given by the following equation [29]:

(8) $$erf\left(x\right)=\frac{1}{\sqrt{\pi }}\int_{-x}^{x}e^{-t^{2}}dt$$

**Figure 1:**
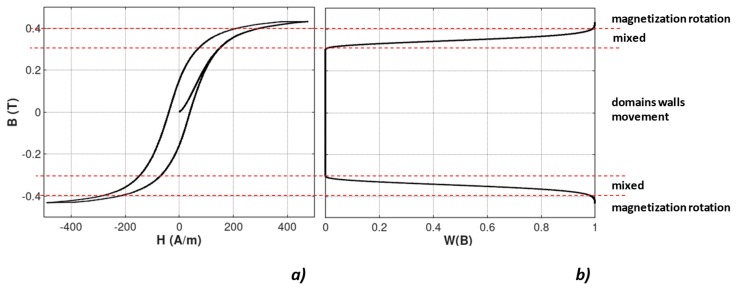
Changes in the mechanism of magnetization in Mn_0.70_Zn_0.24_Fe_2.06_O_4_ ferrite for power applications: (**a**) Magnetic hysteresis loop *B*(*H*); (**b**) *W*(*B*) dependence determining the mechanism of magnetization.

Figure 1 presents the example of the magnetic hysteresis *B*(*H*) loop together with *W*(*B*) dependence. The region of the domain wall movement, the region of the domains magnetization rotation, as well as the region of mixed mechanism can be clearly observed.

Finally, the differential equation determining the flux density *B* dependence of anhysteretic magnetostriction *λ_anhyst_* is stated as:

(9) $$\frac{d\lambda_{anhyst}}{dB}=\frac{d\lambda_{mov}}{dB}\left(1-W\left(B\right)\right)+\frac{d\lambda_{rot}}{dB}W\left(B\right)$$

with the initial condition *λ_anhyst_*(*B*) = 0 for *B* = 0.

**Figure 2:**
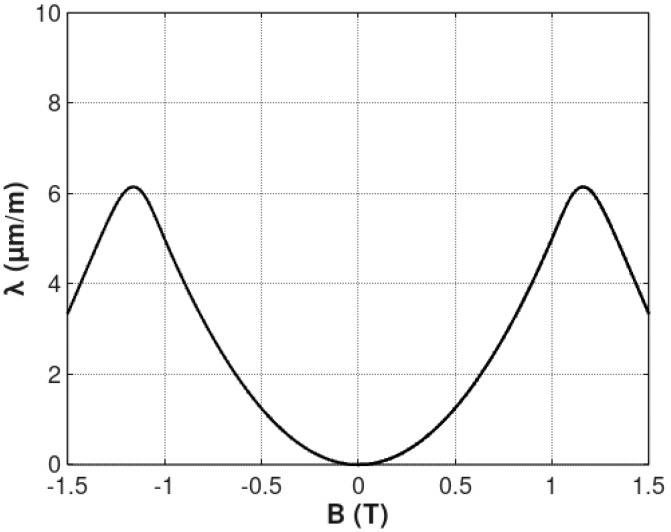
The flux density *B* dependence of anhysteretic magnetostriction *λ_anhyst_*, determined by Equation (9). Parameters: *a*_1_ = 5 $\frac{\mu m}{m}\frac{1}{T^{2}}$, *a*_2_ = 10 $\frac{\mu m}{m}\frac{1}{T}$, *B_switch_* = 1 T, k = 0.1.

The example of the *λ_anhyst_*(*B*) curve stated by Equation (9) is presented in Figure 2. As in the case of real samples, the maximal value of magnetostriction *λ_max_* is significantly higher than saturation magnetostriction *λ_s_*.

The solution proposed to model the “lift-off” phenomenon is similar to the one proposed by Sablik et al. [11,20]. Value of the magnetostriction component *λ_lift-off_* describing this phenomenon is proportional to flux density *B*, if the value of flux density *B* is higher than the value of flux density *B_prev_* reached previously by the material. Otherwise, component *λ_lift-off_* remains unchanged, as the last reached value. Such mechanism describes the physical background of “lift-off” phenomenon, where the magnetostriction component *λ_lift-off_* is connected with the interaction of the magnetostriction strain with the residual stresses in the material. The magnetostriction component *λ_lift-off_* is determined by the following set of differential equations [11]:

(10) $$\frac{d\lambda_{lift-off}}{dB}=a_{lift-off}\;for\;B>B_{prev}\;\frac{d\lambda_{lift-off}}{dB}=\;0\;otherwise$$

**Figure 3:**
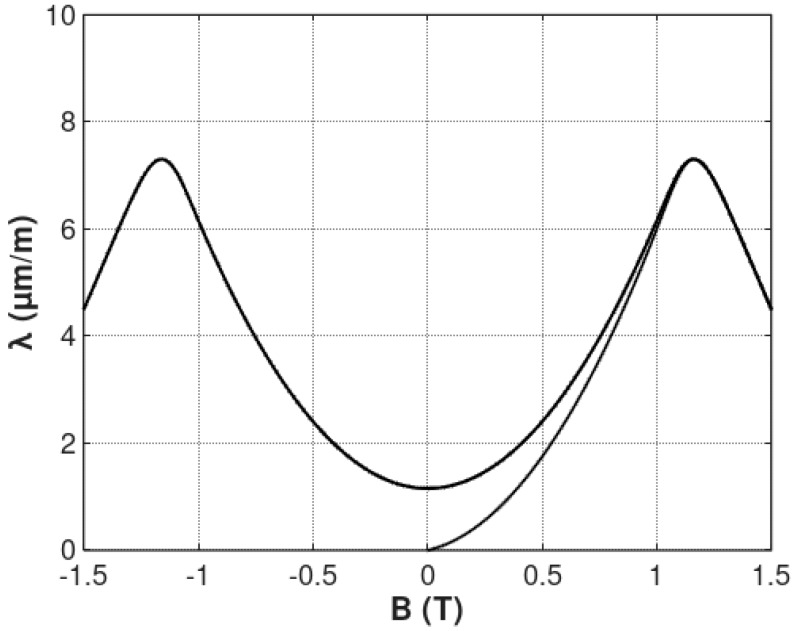
The flux density *B* dependence of anhysteretic magnetostriction *λ_anhyst_* considering the “lift-off” phenomenon, calculated for the following parameters: *a*_1_ = 5 $\frac{\mu m}{m}\frac{1}{T^{2}}$, *a*_2_ = 10 $\frac{\mu m}{m}\frac{1}{T}$, *B_switch_* = 1 T, k = 0.1, *a_lift-off_* = 1 $\frac{\mu m}{m}\frac{1}{T}.$

The example of flux density *B* dependence of anhysteretic magnetostriction *λ_anhyst_* considering the “lift-off” phenomenon is presented in Figure 3.

**Figure 4:**
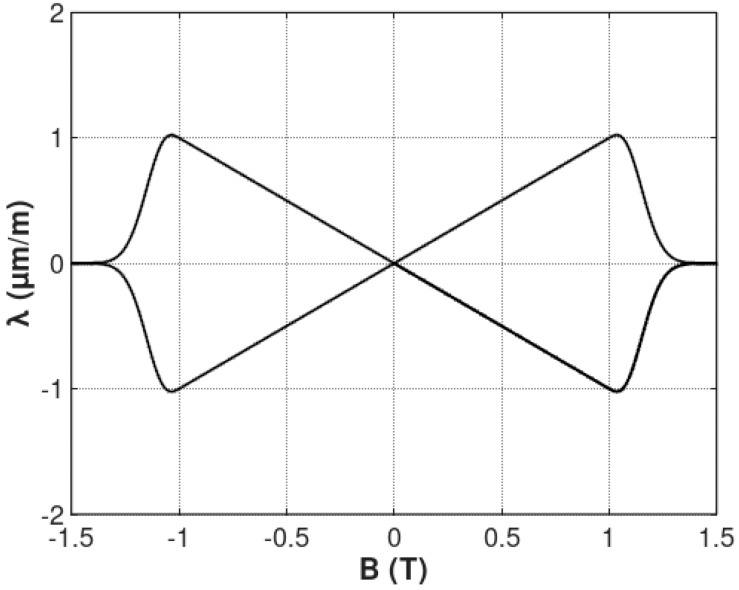
The flux density *B* dependence of magnetostrictive hysteresis *λ_hyst_* calculated for the following parameters: *B_switch_* = 1 T, k = 0.1, *a_hyst_* = 1 $\frac{\mu m}{m}\frac{1}{T}.$

For modelling the hysteretic behavior of magnetostriction *λ*(*B*) dependence, it should be highlighted that hysteresis is connected with the domain walls movement magnetization mechanism. Domain magnetization rotation is anhysteretic, which is what can be observed on both *B*(*H*) and *λ*(*B*) dependencies. As a result, the hysteretic component of the magnetostriction hysteresis loop *λ_hyst_* can be described as:

(11) $$\lambda_{hyst}\left(B\right)=\left(1-W\left(B\right)\right)\cdot a_{hyst}B$$

where *a_hyst_* determines the magnetostrictive hysteresis. The flux density *B* dependence of magnetostrictive hysteresis *λ_hyst_* is presented in Figure 4.

Finally, the *λ*(*B*) hysteresis loop can be calculated as a sum of the components of magnetostriction:

(12) $$\lambda \left(B\right)=\lambda_{anhyst}\left(B\right)+\lambda_{lift-off}\left(B\right)+\lambda_{hyst}\left(B\right)$$

**Figure 5:**
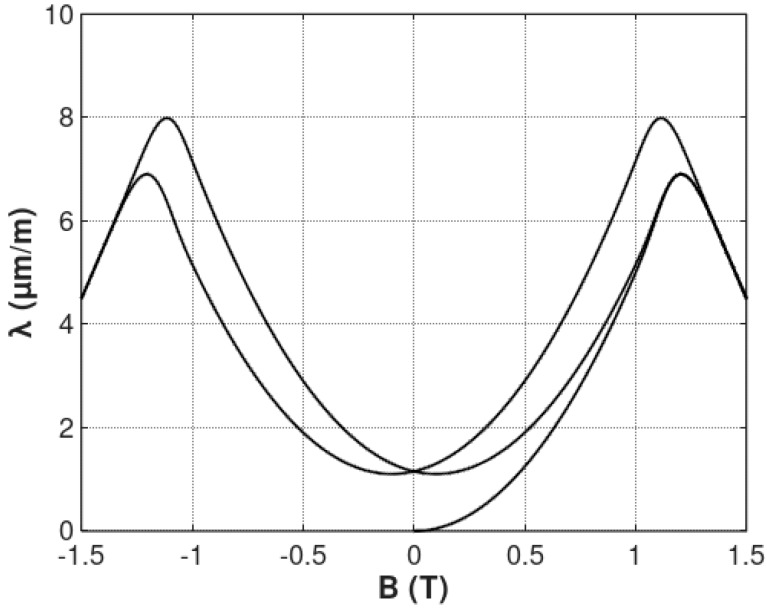
Magnetostrictive *λ*(*B*) hysteresis loop, determined by Equation (12), calculated for the parameters: *a*_1_ = 5 $\frac{\mu m}{m}\frac{1}{T^{2}}$, *a*_2_ = 10 $\frac{\mu m}{m}\frac{1}{T}$, *B_switch_* = 1 T, k = 0.1, *a_lift-off_* = 1 $\frac{\mu m}{m}\frac{1}{T}$, *a_hys_* = 1 $\frac{\mu m}{m}\frac{1}{T}.$

The example of the magnetostrictive *λ*(*B*) hysteresis loop is presented in Figure 5. It can be observed that the shape of the *λ*(*B*) hysteresis loop well reflects the shape of such loops presented in the literature.

## 4. Validation of the Model

### 4.1. Materials and the Method of Measurements

The proposed model was verified on the basis of experimental measurements of magnetostrictive hysteresis loops of Mn_0.70_Zn_0.24_Fe_2.06_O_4_ ferrite for power application [12] and 13CrMo4-5 construction steel [14]. Measurements of the magnetostrictive hysteresis loop were carried out on the frame-shaped samples [30] with strain gauges. Semiconductor strain gauges and foil strain gauges were used for the measurements of the samples made of Mn_0.70_Zn_0.24_Fe_2.06_O_4_ ferrite and 13CrMo4-5 construction steel, respectively. Measurements were performed in room temperature in the quasi-static mode, with the magnetizing field frequency of 0.2 Hz.

**Figure 6:**
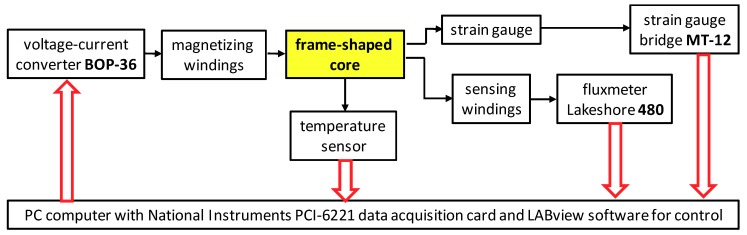
Schematic diagram of the system used for the measurements of magnetostrictive *λ*(*B*) and *λ*(*H*), as well as the magnetic *B*(*H*) hysteresis loops.

A schematic block diagram of the magnetostriction measuring system [12] is presented in Figure 6. Magnetizing winding of the frame-shaped sample was connected to the voltage-current converter BOP36-6M produced by Kepco, USA. The sensing winding as connected to fluxmeter type 480, produced by Lakeshore, USA, enabling real-time measurements of flux density in the core. The strain gauges were connected to a specialized MT-12 bridge, enabling strain gauge sensitivity adjustments. During the measurements, the temperature of the sample was monitored by the thermocouple-based sensor. The whole system was controlled by PC computer, with LabView software equipped in PCI-6221 data acquisition and control card.

### 4.2. Identification of the Parameters of the Model

The proposed model of the magnetostrictive *λ*(*B*) hysteresis loop, stated by Equations (5)–(12), was implemented in Octave 4.4.1, open-source Matlab alternative. For solving the ordinary differential equations, stating the model of themagnetostrictive hysteresis loop, the fourth order Runge–Kutta algorithm was used [31]. Identification of the model’s parameters was carried out on the basis of the minimization process, performed by a derivative-free Nelder and Mead simplex algorithm [32]. The target function *G* for minimization was determined as the sum of the squared differences between the experimental data and the results of the modeling given by the following equation [33]:

(13) $$G=\overset{n}{\underset{i=1}{\sum }}{\left(\lambda_{meas}\left(B_{i}\right)-\lambda_{model}\left(B_{i}\right)\right)}^{2}$$

where *λ_meas_*(*B_i_*) is the results of measurements of the magnetostrictive hysteresis loop for the set of given values of flux density *B_i_*; and *λ_model_*(*B_i_*) is the results of the modeling of the magnetostrictive hysteresis loop for the same set of flux density *B_i_* values.

**Figure 7:**
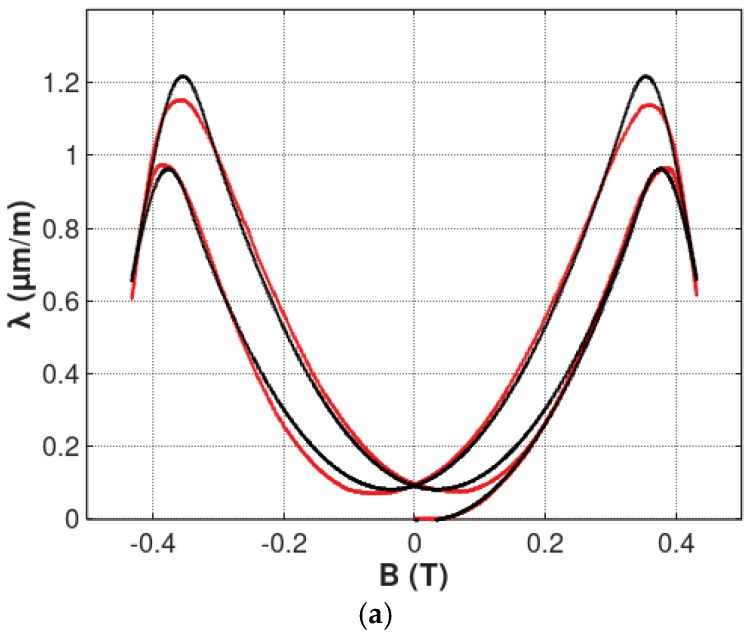

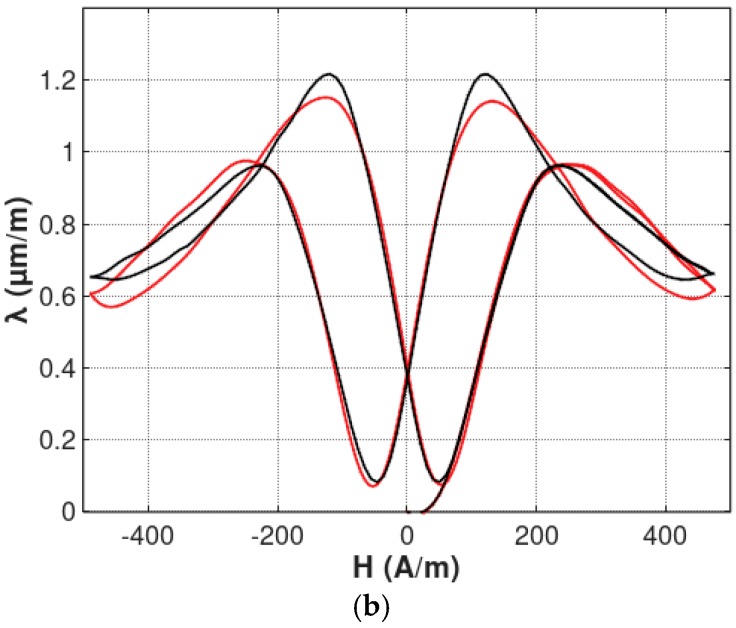
Results of modeling the magnetostrictive hysteresis loops of Mn_0.70_Zn_0.24_Fe_2.06_O_4_ ferrite for power applications: (**a**) *λ*(*B*) hysteresis loop; (**b**) *λ*(*H*) hysteresis loop. Experimental results—red line; results of modeling—black line.

**Figure 8:**
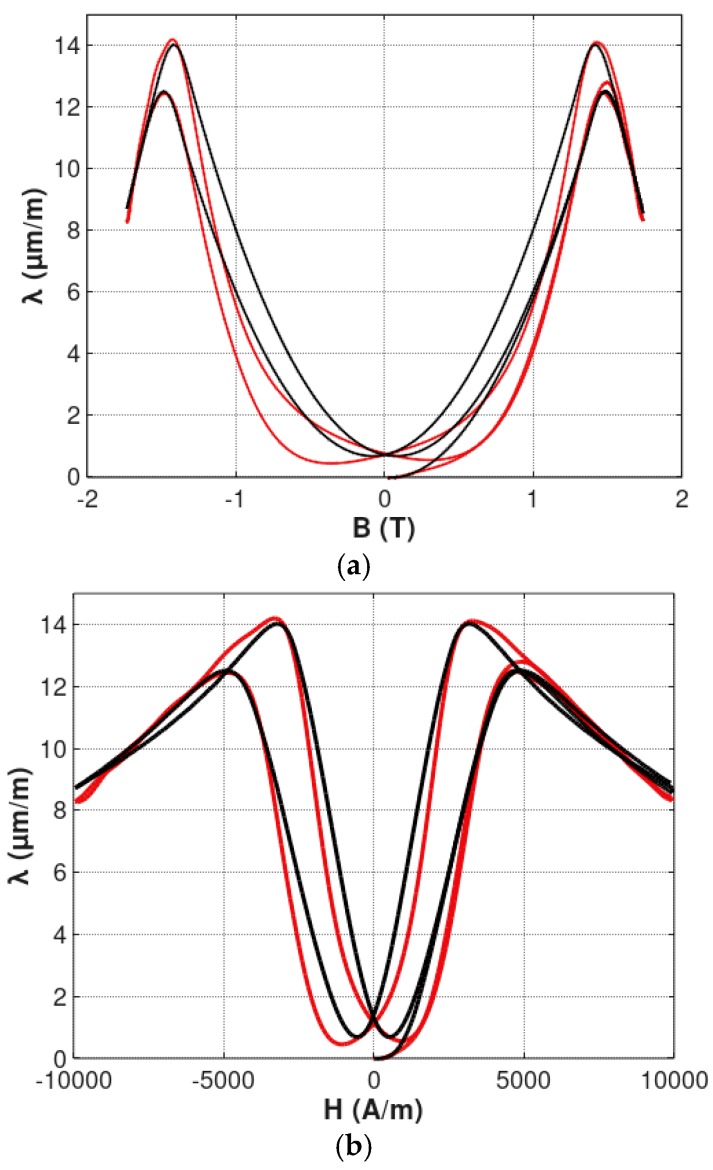
Results of modeling the magnetostrictive hysteresis loops of 13CrMo4-5 steel: (**a**) *λ*(*B*) hysteresis loop; (**b**) *λ*(*H*) hysteresis loop. Experimental results—red line; results of modeling—black line.

Parameters of the magnetostrictive hysteresis loops of Mn_0.70_Zn_0.24_Fe_2.06_O_4_ ferrite for power application and 13CrMo4-5 construction steel, identified during the optimization process, are presented in the Table 1. The results of this modeling are presented in Figure 7 and Figure 8 for Mn_0.70_Zn_0.24_Fe_2.06_O_4_ ferrite, for power application and 13CrMo4-5 construction steel, respectively. Figures present both *λ*(*B*) and *λ*(*H*) hysteresis loops.

It should be highlighted, that the proposed model very well described the shape of both magnetostrictive *λ*(*B*) and *λ*(*H*) hysteresis loops. The quality of the model was also confirmed by the value of the *R^2^* determination coefficient, which describes the proportion of the variance described by the model and the variance of the results of measurement. For *λ*(*B*) hysteresis loops, the R^2^ coefficient exceeded 0.995 and 0.985, in the case of Mn_0.70_Zn_0.24_Fe_2.06_O_4_ ferrite, for power application and 13CrMo4-5 steel, respectively. Such high values of *R^2^* determination coefficients confirms, quantitatively, that the proposed model is suitable for modeling *λ*(*B*) and *λ*(*H*) magnetostrictive hysteresis loops with local maxima. On the other hand, accuracy of the model for 13CrMo4-5 steel is worse than for Mn_0.70_Zn_0.24_Fe_2.06_O_4_ ferrite. This effect is probably connected with the fact that the presented model does not consider nucleation and annihilation mechanisms in the sample. These phenomena should be the subject of further research.

## 5. Conclusions

The presented results of the measurements of both magnetostrictive *λ*(*B*) and *λ*(*H*) hysteresis loops of Mn_0.70_Zn_0.24_Fe_2.06_O_4_ ferrite, for power application and steel 13CrMo4-5, confirm that, in the case of these materials, local maxima occur on the magnetostrictive hysteresis loop. To model such a magnetostrictive hysteresis loop the, change of magnetization and magnetostriction mechanisms should be considered.

The proposed model utilizes the cumulative distribution function of Maxwell–Boltzmann statistical distribution to quantify the transition from domain walls movement to domain magnetization rotation. As a result, a new model of the magnetostrictive hysteresis loop was presented, considering previously proposed descriptions of the magnetostrictive hysteresis and magnetostrictive “lift-off” phenomenon. The proposed model very well describes experimental results, efficiently reproducing the maxima on the magnetostrictive *λ*(*B*) and *λ*(*H*) hysteresis loops. The high quality of the model was confirmed, quantitatively, by the value of the *R^2^* determination coefficient, which exceeded 0.995 and 0.985, for Mn_0.70_Zn_0.24_Fe_2.06_O_4_ ferrite, for power application and 13CrMo4-5 steel, respectively.

Due to good agreement with the experimental results, the proposed model may be used for works focused on understanding the magnetostrictive phenomena in steels and soft ferrites. In addition, the proposed model may be used for the modeling of the magnetostrictive behavior of inductive components with inductive cores made of soft magnetic materials with non-monotonous magnetostrictive characteristics. Such components are commonly used in power conversion devices, such as switching-mode power supplies.

## Figures and Tables

**Table 1:** Parameters of magnetostrictive *λ*(*B*) hysteresis loops determined in optimization process.

Parameter	Unit	Mn_0.70_Zn_0.24_Fe_2.06_O_4_ Ferrite	13CrMo4-5 Steel
*a*_1_	$\frac{\mu m}{m}\frac{1}{T^{2}}$	8.10	6.73
*a*_2_	$\frac{\mu m}{m}\frac{1}{T}$	−10.07	−25.93
*B_switch_*	T	0.312	1.338
*k*	-	0.039	0.101
*a_lift-off_*	$\frac{\mu m}{m}\frac{1}{T}$	0.253	0.495
*a_hyst_*	$\frac{\mu m}{m}\frac{1}{T}$	0.561	0.908
*R* ^2^	-	0.995	0.985

## References

[1] Seco F., Martin J.M., Jimenez A.R. (2009). Improving the Accuracy of Magnetostrictive Linear Position Sensors. IEEE Trans. Instrum. Meas..

[2] Li Y., Sun L., Jin S., Sun L.B. Development of Magnetostriction Sensor for on-line Liquid Level and Density Measurement. Proceedings of the 6th World Congress on Intelligent Control and Automation.

[3] Chang H.-C., Liao S.-C., Hsieh H.-S., Wen J.-H., Lai C.-H., Fang W. (2016). Magnetostrictive type inductive sensing pressure sensor. Sens. Actuators A Phys..

[4] Li P., Liu Q., Li S., Wang Q., Zhang D., Li Y. (2017). Design and numerical simulation of novel giant magnetostrictive ultrasonic transducer. Res. Phys..

[5] Zhu W., Bian L.X., Cheng L., Rui X.T. (2017). Non-linear compensation and displacement control of the bias-rate-dependent hysteresis of a magnetostrictive actuator. Precis. Eng..

[6] Bieńkowski A., Kulikowski J. (1980). The magneto-elastic Villari effect in ferrites. J. Magn. Magn. Mater..

[7] Li M., Li J., Bao X., Mu X., Gao X. (2017). Magnetostrictive Fe_82_Ga_13.5_Al_4.5_ wires with large Wiedemann twist over wide temperature range. Mater. Des..

[8] Zhukov A., Ipatov M., Churyukanova M., Talaat A., Blanco J.M., Zhukova V. (2017). Trends in optimization of giant magnetoimpedance effect in amorphous and nanocrystalline materials. J. Alloys Compd..

[9] Dimitropoulos P.D., Avaritsiotis J.N. (2001). A micro-fluxgate sensor based on the Matteucci effect of amorphous magnetic fibers. Sens. Actuators A Phys..

[10] Dapino M.J. (2004). On magnetostrictive materials and their use in adaptive structures. Struct. Eng. Mech..

[11] Sablik M.J., Jiles D.C. (1988). A model for hysteresis in magnetostriction. J. Appl. Phys..

[12] Bieńkowski A., Szewczyk R. (2018). Magnetostrictive Properties of Mn_0.70_Zn_0.24_Fe_2.06_O_4_ Ferrite. Materials.

[13] Juś A., Nowak P., Gińko O. (2017). Assessment of the Magnetostrictive Properties of the Selected Construction Steel. Acta Phys. Pol. A.

[14] Gińko O., Juś A., Szewczyk R., Szewczyk R., Zieliński C., Kaliczyńska M. (2017). Test Stand for Measuring Magnetostriction Phenomena Under External Mechanical Stress with Foil Strain Gauges. Advances in Intelligent Systems and Computing, Proceedings of the Challenges in Automation, Robotics and Measurement Techniques (ICA 2016), Warsaw, Poland, 2–4 March 2016.

[15] Bozorth R.M. (1978). Ferromagnetism.

[16] Jiles D.C. (2015). Introduction to Magnetism and Magnetic Materials.

[17] Calkins F.T., Smith R.C., Flatau A.B. (2000). Energy-based hysteresis model for magnetostrictive transducers. IEEE Trans. Magn..

[18] Hsu C.-H., Huang Y.-M., Hsieh M.-F., Fu C.-M., Adireddy S., Chrisey D.B. (2017). Transformer sound level caused by core magnetostriction and winding stress displacement variation. AIP Adv..

[19] Cheng S.-J., Liu J.-J., Chang Y.-H., Fu C.-M., Hsu C.-H., Lee C.-Y., Chang C.-W. (2015). Correlation of magnetostriction variation on magnetic loss and noise for power transformer. J. Appl. Phys..

[20] Sablik M.J., Jiles D.C. (1993). Coupled magnetoelastic theory of magnetic and magnetostrictive hysteresis. IEEE Trans. Magn..

[21] Bieńkowski A., Kaczkowski Z. (2000). Major and minor magnetostriction hysteresis loops of Co–Cu–Ni ferrite. J. Magn. Magn. Mater..

[22] Szewczyk R., Bieńkowski A., Kolano-Burian A. (2004). Magnetostrictive properties of Fe_40_Ni_38_Mo_4_B_18_ alloy. Mater. Sci. Eng. A.

[23] Szewczyk R. (2006). Modelling of the magnetic and magnetostrictive properties of high permeability Mn-Zn ferrites. PRAMANA J. Phys..

[24] Ueda Y., Takahashi M. (1988). Structure and magnetic properties in single-crystal iron film electrodeposited on a (110) copper crystal. J. Magn. Magn. Mater..

[25] Gou J., Liu X., Wu K., Wang Y., Hu S., Zhao H., Xiao A., Ma T., Yan M. (2016). Tailoring magnetostriction sign of ferromagnetic composite by increasing magnetic field strength. Appl. Phys. Lett..

[26] Bozorth R.M., Tilden E.F., Williams A.J. (1955). Anisotropy and Magnetostriction of Some Ferrites. Phys. Rev..

[27] Piotrowski L., Chmielewski M., Augustyniak B. (2016). On the correlation between magnetoacoustic emission and magnetostriction dependence on the applied magnetic field. J. Magn. Magn. Mater..

[28] Tremolet E. (1992). Magnetostriction.

[29] Mandl F. (2008). Statistical Physics.

[30] Bieńkowski A. (1992). Some problems of measurement of magnetostriction in ferrites under stresses. J. Magn. Magn. Mater..

[31] Atkinson K.A. (1989). An Introduction to Numerical Analysis.

[32] Nelder J.A., Mead R. (1965). A simplex method for function minimization. Comput. J..

[33] Biedrzycki R., Jackiewicz D., Szewczyk R. (2014). Reliability and Efficiency of Differential Evolution Based Method of Determination of Jiles-Atherton Model Parameters for X30Cr13 Corrosion Resisting Martensitic Steel. J. Autom. Mob. Robot. Intell. Syst..

